# Induction of Glutathione Synthesis Provides Cardioprotection Regulating NO, AMPK and PPARa Signaling in Ischemic Rat Hearts

**DOI:** 10.3390/life11070631

**Published:** 2021-06-29

**Authors:** Yulia V. Goshovska, Raisa A. Fedichkina, Volodymyr V. Balatskyi, Oksana O. Piven, Pawel Dobrzyn, Vadym F. Sagach

**Affiliations:** 1 Department of Blood Circulation, Bogomoletz Institute of Physiology, National Academy of Sciences of Ukraine, 4 Bogomolets Str., 01024 Kyiv, Ukraine; fedichkina@biph.kiev.ua (R.A.F.); vsagach@biph.kiev.ua (V.F.S.); 2 Laboratory of Molecular Medical Biochemistry, Nencki Institute of Experimental Biology, Polish Academy of Sciences, 3 Pasteur Str., 02-093 Warsaw, Poland; v.balatskyi@nencki.edu.pl (V.V.B.); p.dobrzyn@nencki.edu.pl (P.D.); 3 Department of Human Genetics, Institute of Molecular Biology and Genetics, National Academy of Sciences of Ukraine, 150 Akad. Zabolotnogo Str., 03680 Kyiv, Ukraine; o.o.piven@imbg.org.ua

**Keywords:** cardioprotection, glutathione, ischemia, heart, SERCA, AMPK, PPARα

## Abstract

Glutathione (GSH) is essential for antioxidant defence, and its depletion is associated with tissue damage during cardiac ischemia-reperfusion (I/R). GSH is synthesized by the glutamate-cysteine ligase enzyme (GCL) from L-cysteine, which alternatively might be used for hydrogen sulfide production by cystathionine-gamma-lyase (CSE). Here, we have investigated whether in vivo treatment with L-cysteine and an inhibitor of CSE,D,L-propargylglycine (PAG), can modulate cardiac glutathione and whether this treatment can influence heart resistance to I/R in a Langendorff isolated rat hearts model. Pretreatment with PAG + L-cysteine manifested in pronounced cardioprotection, as there was complete recovery of contractile function; preserved constitutive NOS activity; and limited the production of reactive oxygen and nitrogen species in the ischemized myocardium. Cardiac GSH and GSSG levels were increased by 3.5- and 2.1-fold in PAG + L-cysteine hearts and were 3.3- and 3.6-fold higher in PAG + L-cysteine + I/R compared to I/R heart. The cardioprotective effect of PAG + L-cysteine was completely abolished by an inhibitor of GCL, DL-buthionine-(S,R)-sulfoximine. Further analysis indicated diminished fatty acid β-oxidation, increased glucose consumption and anaerobic glycolysis, and promoted OXPHOS proteins and SERCA2 in PAG + L-cysteine + I/R compared to the I/R group. PAG + L-cysteine inhibited PPARα and up-regulated AMPK signalling in the heart. Thus, induction of glutathione synthesis provided cardioprotection regulating NO, AMPK and PPARa signaling in ischemic rat hearts.

## 1. Introduction

Ischemia/reperfusion (I/R) causes cell damage through many mechanisms that involve oxidative stress, depletion of energy substrates, calcium overload, uncoupling of nitric oxide syntase (NOS), etc. Many signalling pathways are activated during I/R heart remodelling. AMP-activated proteinkinase (AMPK) is avital regulator of cell survival, activated in response to cellular adenosine nucleotides (AMP, ADP, or ATP) and intensive reactive oxygen species (ROS) production [1]. ROS have the ability to modulate the activity of crucial regulators of cardiac biology, such as canonical WNT, Notch, PI3K/AKT and other signalling pathways [2,3]. This signalling governs cell apoptosis and survival, mitochondrial function and metabolic plasticity of cardiomyocytes in the injured heart [4].

In ischemia, drastic lack of oxygen induces a metabolic switch from aerobic ATP production to glycolysis [5]; thus, ATP deficiency and impairment of ATP-dependent transporters, channels and carriers, including sarcoplasmic/endoplasmic reticulum calcium ATP-ase (SERCA), occurs. Calcium overload and burst production of ROS in the first minutes of reperfusion mediate irreversible opening of mitochondrial permeability transition pores and cardiomyocytes death [6,7]. In conditions of I/R, oxidative stress is accompanied with uncoupling of constitutive NOS and generation of superoxide anion radicals instead of nitric oxide [8]. Therefore, attenuation of massive ROS production, preservation of mitochondrial membrane integrity and re-coupling of NOS are needed for successful cardioprotection.

As a consequence of extensive ROS production, activation of antioxidant defence occurs. The reduced form of glutathione (GSH) is one of the most essential antioxidant molecules in the heart due to its ability to scavenge ROS. Conjugation of glutathione with a cysteine thiol group on the target proteins is a redox-mediated post-translational modification that affects metabolism in many physiological and pathological conditions. S-glutathionylation of SERCA and ryanodine receptors lead to the activation of these proteins improving excitation–contraction coupling in cardiomyocytes [9,10], whereas S-glutathionylation of NOS leads to NOS uncoupling and a decrease in NO bioavailability in oxidative stress [11]. Glutathione is a cofactor for a number of enzymes that provide antioxidant protection. Glutathione transferase uses glutathione to neutralize xenobiotics and reduces hydroperoxides to alcohols. Glutathione peroxidase reduces hydrogen peroxide to water and oxidizes GSH to the oxidized form (GSSG).

The ratio of GSH/GSSGhas been shown as an important index for the redox state of the cell [12]. It was shown that GSH levels were decreased in heart tissue after myocardial infarction [13,14]. In hypertensive patients, the levels of GSH as well as glutathione reductase and glutathione peroxidase activities were decreased [15]. Depletion of GSH in mitochondria might be the main cause of cytochrome oxidase C oxidation and the development of mitochondrial dysfunction in I/R [16]. It has been shown that recovery of cardiac function after experimental myocardial infarction was much worse in mice with glutathione peroxidase deficiency compared with wild-type mice, whereas overexpression of glutathione peroxidase prevented left ventricular failure after myocardial infarction [17]. Thus, this evidence suggests the extreme importance of glutathione in the maintenance of redox status of the heart and myocardial resistance to I/R-induced oxidative stress.

Glutathione is a tripeptide synthesized from the aminoacidscysteine, glutamic acid and glycine. Glutamate-cysteine ligase (GCL) catalyzes the first reaction and is a rate-limiting enzyme in glutathione synthesis. Few reports showed the antioxidative effect of L-cysteine and its derivatives [18,19,20,21]. Elsey et al. suggested that L-cysteine decreases the infarct zone of the myocardium, acting at least partially as the precursor of hydrogen sulphide (H_2_S) synthesis [21]. However, L-cysteine is a precursor for glutathione as well. We hypothesized that inhibition of L-cysteine conversion to H_2_S and direction of L-cysteine into the glutathione synthesis pathway will have beneficial effects on the myocardial resistance to I/R. For this purpose we used the Langendorff isolated ratheart model for I/R injury study, D,L-propargylglycine (PAG) as an inhibitor of the cystathionine γ-lyase (CSE) enzyme, which produce H_2_S from L-cysteine [22,23], and D,L-buthionine-(S,R)-sulfoximine (BSO) as an inhibitor of GCL [24].

## 2. Materials and Methods

### 2.1. Animals and Treatments

The study was conducted according to the guidelines of the Declaration of Helsinki, Directive 2010/63/EU of the European Parliament and of the Council on the protection of animals used for scientific purposes (22 September 2010) and tothe Law№3447-IV (2006) “On the Protection of Animals from Brutal Treatment”, adopted by the Parliament of Ukraine. It is also approved by the Institutional Ethics Committee of Bogomoletz Institute of Physiology of National Academy of Science of Ukraine (protocol 4/20 from 22.05.20).

The study was conducted on adult (6 months, body weight averaged 250 g) male Wistar rats housed in the vivarium of Bogomoletz Institute of Physiology (Kyiv, Ukraine) on a natural day-to-night cycle with free access to food and water. Animals were randomly assigned to one of five groups: group I (control), group II (treated with PAG 40 min before decapitation), group III (treated with L-cysteine 30 min before decapitation), group IV (treated with PAG and L-cysteine), group V (treated with BSO, PAG and L-cysteine). The doses of PAG and L-cysteine were 11.3 and 121 mg·kg^−1^ respectively. Glutathione inhibitor, BSO, was injected 1 min before PAG administration in a dose of 22.2 mg·kg^−1^. All chemicals (purchased from Sigma-Aldrich Chemie GmbH, Taufkirchen, Germany) were dissolved in 0.3–0.4 mL of sterile water and injected intraperitoneally with separate syringes.

### 2.2. Isolated Hearts by Langendorff Preparation

Hearts were perfused using Langendorff technique. The aorta was cannulated and the perfusion of the coronary vessels of the isolate heart was performed retrogradely under stable perfusion pressure of 75–80 mmHg with non-recirculating Krebs-Henseleit solution (in mmol/L): 118 NaCl, 4.7 KCl, 1.2 MgSO_4_, 24 NaHCO_3_, 1.2 KH_2_PO_4_, 10.0 glucose, 2.5 CaCl_2_, pH 7.4. The perfusing solution was aerated with 95% O_2_/5% CO_2_. Temperature of the perfusion solution was held constant at 37 °C. The pulmonary artery was catheterised with a tube of a proper size for effluent collection. Thus, a coronary flow was measured as the volume of the perfusion solution passed through the heart per minute. The following parameters were assessed: the left ventricular developed pressure (LVDP), calculated as the difference between systolic and diastolic pressure in the left ventricle; the end-diastolic pressure (EDP); the maximal (dP/dt_max_) and minimal (dP/dt_min_) value of the first derivative of the left ventricular pressure, and the heart rate. LVDP was monitored with a water-filled latex balloon inserted into the left ventricle, inflated to obtain a diastolic pressure of 5 to 10 mmHg. The balloon was connected to the strain gauge 746 and policardiograph (‘Mingograph’, ‘Elema’, Stockholm, Sweden) for continuous monitoring and registration of the pressure changes throughout the experiment. After 20 min equilibration, hearts were submitted to I/R protocol (20/40 min), biochemical studies or Western blot (WB) analysis.

### 2.3. Oxygen Consumption by Myocardium of Isolated Rat Heart

The ‘arterial’ samples of perfusion solution were collected from the tap place close to the cannulated aorta. Samples of the ‘venous’ perfusate were collected without exposure to air by inserting the needle of testing syringe into the pulmonary catheter. Oxygen tension of those perfusate samples was measured with the gas analyzer (BMS 3 Mk-2 Radiometer, Copenhagen, Denmark). Arteriovenous difference was calculated and used for oxygen consumption calculation by formula proposed by Neely [25]:

O_2_ consumption (mmoles/hr per g) = arteriovenous O_2_ tension (mmHg)/760 (mmHg) × solubility of O_2_ at 37 °C (ml/ml H_2_O)/22.4 (ml/mmole) × coronary flow (ml/hr)/dry weight of heart (g)

The oxygen cost of myocardial work (OCMW) was expressed as the ratio of oxygen consumption to the heart work (the product of LVDP and the heart rate).

### 2.4. Glutathione Assay

The measurements of the oxidized (GSSG) and the reduced glutathione (GSH) were performed in the heart homogenates with Ellman’s reagent [26]. Hearts were removed from Langendorff apparatus, washed with cold (+4 °C) 0.9% KCl solution, weighed and homogenized in the isolation buffer based on 0.1 M potassium phosphate buffer (KPE) with the addition of 5 mM EDTA, pH = 7.5, 0.1% Triton X-100 and 0.6% sulfosalicylic acid. The ratio of isolation buffer and tissue was 1:9. The homogenized tissue was centrifuged (Allegra X-22R, Beckman Coulter, Brea, California, USA) at +4 °C, 8000× *g* for 10 min. After this stage, the precipitate was leached into clean microtubes. 1 mL of precipitate for GSH measurement was immediately frozen (−20 °C). For GSSG measurement, 1 mL of precipitate was mixed with 30 μL of 97% 2-vinylpyridine in KPE solution (1:10), stirred, and 60 μL of 98% triethanolamine in KPE solution (1:6) was added after an hour, stirred and frozen. The measurements were performed using a BiosanHiPo MPP-96 microplate reader (Lithuania). 60 μL of 500 units glutathione reductase in KPE solution (1:150), 60 μL of 0.8 mmol/L cofactor β-NADPH reduced tetrasodium salt and 60 μL of 1.68 mmol/L dithiobisnitrobenzoic acid were added to initiate the reaction. Optical density was measured immediately and for 2 min each 30 s at 405 nm. The concentrations of GSH and GSSG were calculated according to the linear regression equation obtained from the calibration curve of the standard GSH and GSSG solutions.

### 2.5. Measurement of H_2_O_2_

Protein-free homogenates of heart tissue were added to the quartz cuvette (1 cm) containing 2 mL of 0.1 mol/L KJ, lactoperoxidase excess (50 nmol/L) in 0.05 mol/L phosphate buffer, pH = 7.33. Rapid changes in extinction were recorded at 353 nm. The amount of H_2_O_2_ was expressed in pmol per mg of protein samples using a molar extinction coefficient ε = 26,000 M^−1^ cm^−1^ [27].

### 2.6. Measurement of Superoxide Radical (˙O_2_^−^) Generation Rate

The ˙O_2_^−^ content was measured by oxidation of cytochrome c in 10 mmol/L HCl-Tris buffer (pH 7.4, t = 37 °C) recording changes of absorbance of the sample containing mixture at 550 nm during 30 min. The amount of generated ˙O_2_^−^ was expressed in nmol per mg of protein per min using a molar extinction coefficient ε = 21,000 M^−1^ cm^−1^ [28].

### 2.7. Measurement of Diene Conjugates

The content of diene conjugates was determined spectrophotometrically by optical density at 232 nm in heptanoic extracts of the samples [29]. The amount of dien conugates was determined using molar absorption coefficient ε = 21,000 mol^−1^∙cm^−1^.

### 2.8. Measurement of NOS Activity

To determine the total NO-synthase activity (Ca^2+^-dependent and Ca^2+^-independent) in the heart homogenates, we used a combination of methods [30,31] adapted to the spectrophotometric measurement of L-citrulline as a reaction product. The aliquots of the samples containing 500–1000 mg of protein were incubated in a total volume of 1 mL of a substrate mixture of the following composition (mmol/mL): KH_2_PO_4_—50, MgCl_2_—1, CaCl_2_—2, NADPH—1, L-arginine—2, pH 7.0,for 60 min at 37 °C. The reaction was stopped by adding 0.3 mL of 2N HClO_4_. The mixture was centrifuged at 3500 g/min for 10 min, and L-citrulline content was determined in protein-free supernatant by highly specific spectrophotometric method from color reaction with antipyrine. For inducible NOS (iNOS, Ca^2+^-independent) activity assessment, we used the same method but with addition of 2 mmol/L EDTA in the incubation mixture instead of CaCl_2_. The activity of constitutive NOS (cNOS, Ca^2+^-dependent) was calculated by subtracting that of iNOS from the total NOS activity. The activities of enzymes were expressed in picomols of newly formed L-citrulline for 1 min per 1 mg of protein.

### 2.9. Measurement of Nitrite Content

Nitrite content (NO_2_^−^) was determined in protein-free aliquots from the heart homogenates in a colorimetric reaction [32]. The aliquots of protein-free sample were mixed with Griess reagent in the proportion of 1:1. After 5 min of incubation at room temperature, optical density at 546 nm was measured. The concentration of nitrite was determined from calibration curve build by NaNO_2_.

### 2.10. Measurement of Nitrate Content

Nitrate content was measured by the method based on the reduction of NO_3_^−^ to NO_2_^−^ by zinc [33]. For the determination of the summary content of NO_2_^−^ + NO_3_^−^, 1.0 mL the aliquots of the protein-free probes at pH 7.4 were mixed with 50 μL of zinc suspension (100 mg∙mL^−1^ in water). After 5 min of centrifugation 0.5 mL of the supernatants were sampled and mixed with equal volume of the Griess reagent. Then, optical density at 546 nm was measured. For the determination of nitrate content, that of nitrite, established in parallel missing the reduction procedure, was subtracted.

### 2.11. Measurement of H_2_S

H_2_S content was measured as described previously with modifications [34]. Cardiac tissue was homogenized in a 10-fold volume (*w*/*v*) of 50 mmol/L ice-cold potassium phosphate buffer (pH 6.8), mixed with 0.5 mL of 1% zinc acetate and incubated for 10 min at 37.5 °C. Consequently, 0.5 mL of 20 mmol/L N,N-dimethyl-p-phenylenediamine sulfate in 7.2 mol/L HCl and 0.5 mL of 30 mmol/L FeCl_3_ in 1.2 mol/L HCl were added and incubated for 10 min in a dark and cold place. 0.5 mL 10% trichloroacetic acid was added to the reaction mixture prior to the addition of 2.5 mL distilled water. The optical density of the mixture was measured at 670 mn. The quantity of H_2_S was determined by calibration curve of NaHS.

### 2.12. Measurementof CSE + CBS Activity

The total activity of H_2_S-producing enzymes (CSE + CBS) was estimated by de novo produced H_2_S with the method described previously adding some modifications [34,35]. Myocardial tissue was homogenized in 1:10 (*w*/*v*) 50 mmol/L ice-cold potassium phosphate buffer (pH 6.8). The aliquots of the samples were incubated in medium contained 0.67 mmol/L piridoxalphosphate, 3.3 mmol/L L-cysteine 0.083 mol/L tris buffer (pH 8.5) for 30 min. Then, 0.5 mL 1% zinc acetate was added to trap the newly formed H_2_S. After 90 min at 37 °C, the reaction was terminated with the addition of 0.5 mL 10% trichloroacetic acid. 0.5 mL 20 mmol/L N,N-dimethyl-p-phenylenediamine sulfate in 7.2 mol/L HCl was added immediately, followed by the addition of 0.5 mL 30 mmol/L FeCl_3_ in 1.2 mol/L HCl. In 20 min the optical density of the mixture was measured at 670 nm. The quantity of H_2_S was determined by calibration curve of NaHS. The H_2_S production rate was expressed as picomoles of H_2_S per mg of protein per minute.

### 2.13. Western Blotting

The hearts were homogenized in radioimmunoprecipitation assay buffer as described earlier [36]. Western blot was performed using the following antibodies: β-catenin (1:1000, sc-7963, Santa Cruz Biotechnology, Santa Cruz, CA, USA), active β-catenin (1:1000, 05-665, Millipore, Billerica, MA, USA), glycogen synthase kinase 3α/β (GSK3; 1:1000, 5676, Cell Signaling, Danvers, MA, USA), phosphorylated GSK3α/β at Ser21/9 (pGSK3; 1:1000, 8566, Cell Signaling), Akt1 (1:1000, sc-1618, Santa Cruz Biotechnology), phosphorylated Akt at Ser473 (1:500, sc-101629, Santa Cruz Biotechnology), Erk1/2 (1:1000, 9102, Cell Signaling), phosphorylated Erk1/2 at Thr202/Thr204 (pErk1/2, 1:1000, 4377, Cell Signaling), AMPKα 1/2 (1:500, sc-25792, Santa Cruz Biotechnology), phosphorylated AMPKα at Thr172 (pAMPKα, 1:500, sc-33524-R, Santa Cruz Biotechnology), PKA (1:500, sc-390548, Santa Cruz Biotechnology), phosphorylated PKA (pPKA; 1:500, sc-32,968, Santa Cruz Biotechnology), hormone-sensitive lipase (HSL; 1:1000, 4107, Cell Signaling), phosphorylated HSL (pHSL) at Ser565 (1:1000, 4137, Cell Signaling), acetyl-CoA carboxylase (ACC; 1:1000, 04-322, Millipore), phosphorylated ACC (pACC; 1:500, 07-303, Millipore), proliferator-activated receptor alpha (PPARα; 1:1000, sc-9000, Santa Cruz Biotechnology), GLUT4 (1:100, sc-7938, Santa Cruz Biotechnology), pyruvate dehydrogenase kinase 1 (PDK1, 1:100, sc-7140, Santa Cruz Biotechnology), phosphorylated AS160 (pAS160, 1:1000, 4288, Cell signaling), AS160 (1:1000, 2670, Cell signaling), mammalian target of rapamycin (mTOR, 1:1000, 2983, Cell Signaling), phosphorylated at Ser 2448 mTOR (1:1000, 5536, Cell Signaling), SIRT1 (1:1000, 07-131, Millipore), PGC-1α (1:1000, NBP1-04676, Novus biologicals), OXPHOS (1:1000, ab110413, Abcam), Serca2 (1:1000, sc-376235, Santa Cruz) and GAPDH (1:1000, 5174, Cell Signaling) as a loading control. After exposure to horseradish peroxidase-conjugated secondary antibodies: anti-mouse (1:10,000, sc-2005, Santa Cruz Biotechnology), anti-rabbit (1:10,000, sc-2004, Santa Cruz Biotechnology), anti-goat (1:10,000, sc-2020, Santa Cruz Biotechnology), for 1 h, target proteins were visualized by enhanced chemiluminescence (Pierce, Rockford, IL, USA). Proteins were quantified by densitometry.

### 2.14. Data Analysis

The data were expressed as the mean ± SEM. Shapiro–Wilk test was used to analyze the normality of distribution. Comparison between groups was made using one-way ANOVA with post hoc Tukey HSD test or non-parametric Kruskal-Wallis with Mann-Whitney post hoc analysis. *p* values less than 0.05 were significant.

## 3. Results

### 3.1. L-cysteine Improved Relaxation of Heart after Ischemia

**Figure 1:**
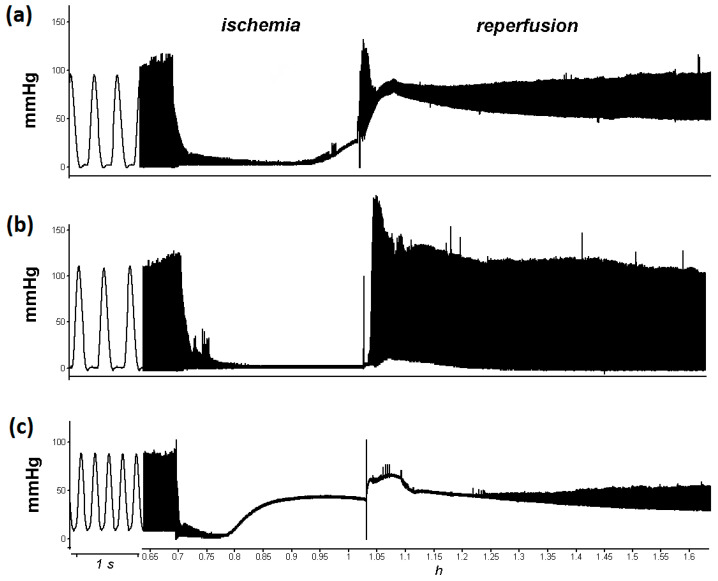
Native records of the left ventricle contractile function of the isolated rat heart during ischemia/reperfusion in control (**a**), in PAG + L-cysteine (**b**) and BSO + PAG + L-cysteine (**c**) groups. Administration of PAG and L-cysteine prevented ischemic contracture of the myocardium and supported complete restoration of contractile activity of the left ventricle, whereas the pretreatment with BSO intensified ischemic contracture of the myocardium and abolished protective effect of PAG + L-cysteine.

Cardiac contractility was greatly depressed in 20 min of total ischemia and reperfusion in control group (Figure 1a).

**Figure 2:**
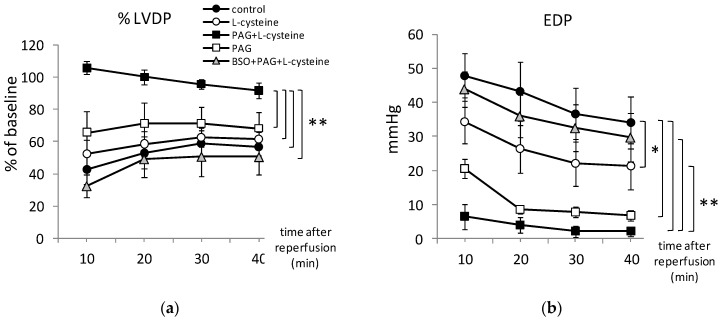

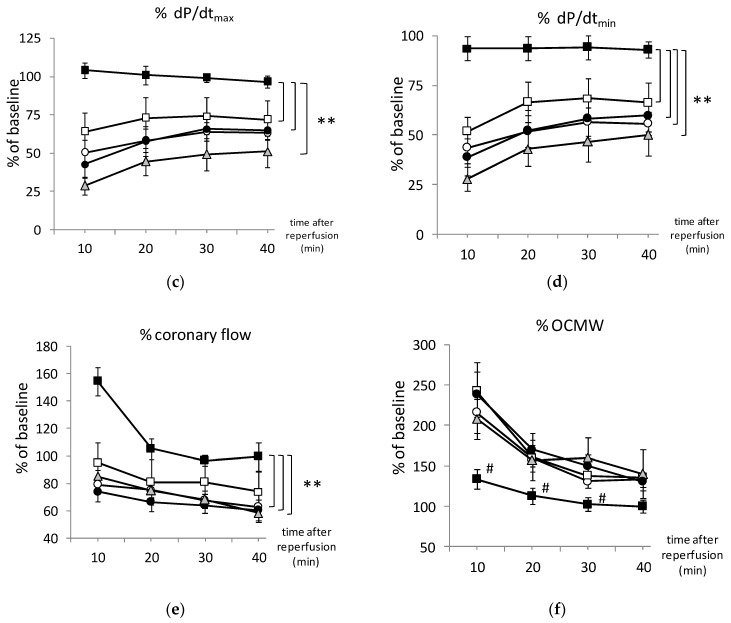
Effect of L-cysteine and inhibitors of its conversion to H_2_S (by PAG) or to glutathione (by BSO) at restoration of cardiodynamics of the isolated rat heart after 20 min of total ischemia: the left ventricular developed pressure (LVDP) (**a**), the end-diastolic pressure (EDP) (**b**), the maximal rate of rise of the left ventricular pressure,dP/dt_max_ (**c**), the minimax rate of rise of the left ventricular pressure, dP/dt_min_ (**d**), the coronary flow (**e**), the oxygen cost of myocardial work (**f**). *n* = 4 to 8 in each group. * *p* < 0.05, ** *p* < 0.01 as calculated by Kruskal-Wallis with Mann-Whitney post hoc analysis; ^#^ *p* < 0.05 compared to the control group at the same time point as calculated by Kruskal-Wallis with Mann-Whitney post hoc analysis.

The hearts subjected to I/R exhibited considerably impaired relaxation. Specifically, an intensive contracture was observed during ischemia. At the end of ischemia, the value of EDP averaged 48 mmHg (Figure 2b). At the 40th min of reperfusion, LVDP (Figure 2a) as well as coronary flow (Figure 2e) were restored only to 59% compared to the pre-ischemic values. During all the period of reperfusion, dP/dt_max_ as well as dP/dt_min_ were decreased twice (Figure 2c,d). Post-ischemic disturbances of heart function were accompanied with the changes in oxygen metabolism. At the 10th min of reperfusion, OCMW was 240%, if compared to the pre-ischemic values and dropped to 130%at the 40th min of reperfusion, thus indicating ineffective oxygen utilization by the ischemized myocardium (Figure 2f).

In L-cysteine treated group, LVDP was 52.5% ± 15.5% and 61.6% ± 6.5% at the 10th and the 40th min of reperfusion respectively (Figure 2a). Dynamic of dP/dt and coronary flow restoration did not differ significantly from the control group. However, increase of EDP was significantly prevented by L-cysteine indicating improved relaxation of the ischemized myocardium (Figure 2b). L-cysteine slightly decreased OCMW that averaged 216.1% ± 23.5% and 133.0% ± 6.8 at the 10th and the 40th min of reperfusion respectively (Figure 2f). These results suggest that pretreatment with L-cysteine diminished post-ischemic disturbances of the heart function and oxygen metabolism.

### 3.2. PAG + L-Cysteine Pretreatment Improved Heart Function Recovery and Preserved SERCA2 Level after Ischemia

**Figure 3:**
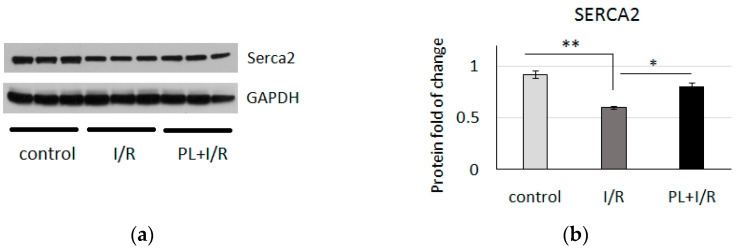
PAG + L-cysteine (PL) pretreatment preserves SERCA2 level after ischemia/reperfusion. (**a**) Western blot of SERCA2in heart lysates from the control, I/R and PAG + L-cysteine + I/R (PL + I/R) hearts. (Original western blot see supplementary Figure S1) (**b**) Densitometry of SERCA2 protein normalized to GAPDH. *n* = 6 to 8 in each group. The control hearts were perfused by Langendorff preparation about 20 min, I/R hearts underwent 20 min of global ischemia followed by 40 min of reperfusion. Then, lysates for WB were prepared. * *p* < 0.05, ** *p* < 0.01 as calculated by one-way ANOVA with post hoc Tukey HSD test.

The ischemic contracture was not observed in any experiment in PAG + L-cysteine group (Figure 1b). Notable prevention of post-ischemic disturbances of all monitored parameters was observed. At the 10th min of reperfusion, LVDP restored to 106% ± 3.8% compared to 42.7% ± 21.3% in the control group (*p* < 0.01) (Figure 2a). EDP averaged 5 mmHg during reperfusion (Figure 2b). It is important to note that improved relaxation of the left ventricle was supported by analysis of SERCA2 protein level, which was increased in heart lysates of PAG + L-cysteine + I/R group compared to I/R hearts (Figure 3). This might indicate sufficient ATP supply and, thus, improved Ca^2+^ re-entry into sarcoplasmic reticulum in the ischemized myocardium after PAG + L-cysteine pretreatment.

Post-ischemic recovery of the cardiac contractility was greatly improved in PAG + L-cysteine group: at the 10th min of reperfusion, dP/dt_max_ was 104.0% ± 3.0% and dP/dt_min_ was 93.5% ± 4.0% compared to 42.8% ± 20.7% and 38.8% ± 24.2% respectively in the control group (Figure 2c,d). The improvement of the contractile function was not deteriorated to the end of reperfusion. Dynamics of coronary flow restoration differed significantly from other groups. At the 10th min of reperfusion, OCMW was significantly lower than in control group and increased only by 34% with subsequent decrease (Figure 2f). These data clearly indicate more effective oxygen utilization by cardiac tissues under PAG + L-cysteine pretreatment compared to the control group.

To confirm that the observed cardioprotection was caused by PAG + L-cysteine pretreatment we performed the experiments with PAG administration only. It is interesting that PAG pretreated hearts demonstrated significantly lower EDP values (Figure 2b) and strong tendency to improved LVDP and dP/dt upon reperfusion. We revealed neither difference in coronary flow between PAG and PAG + L-cysteine group nor between PAG and control group (Figure 2e). The dynamic of OCMW repeated the values of control group (Figure 2f). Thus, PAG in examined dose improved cardiac relaxation after I/R but combination of PAG and L-cysteine provided pronounced cardioprotective effect.

### 3.3. PAG + L-Cysteine Pretreatment Induced Glutathione Synthesis

We hypothesized that one of the possible mechanisms of cardioprotective effect of PAG + L-cysteine may be induced by the engagement of L-cysteine in de novo formation of glutathione. To test this assumption, we performed experiments with the inhibitor of glutathione, BSO. Our results clearly demonstrated that BSO completely abrogated cardioprotective effect of PAG + L-cysteine (Figure 1c). The restoration of cardiodynamic parameters in BSO + PAG + L-cysteine group differed significantly from PAG + L-cysteine group upon the reperfusion whereas dynamic of OCMW repeated the pattern of control group (Figure 2).

**Figure 4:**
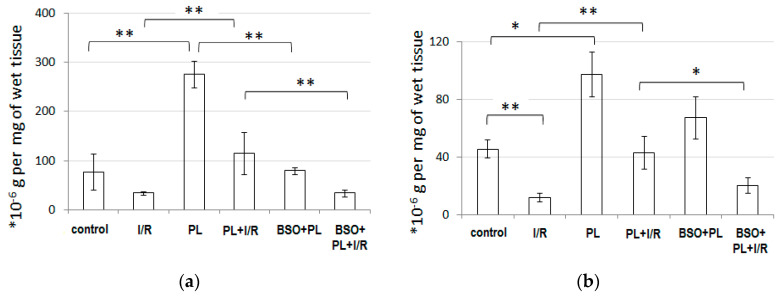
GSH (**a**) and GSSG (**b**) levels in the heart homogenates. Rats were treated with propargyl glycine and L-cysteine (PL) or with D,L-buthionine-(S,R)-sulfoximine +PL (BSO + PL), and then isolated hearts were submitted to Langendorffpreparation and underwent 20 min of global ischemia followed by 40 min of reperfusion (I/R). Control hearts were perfused by Langendorff preparation about 20 min. Then, probes for glutathione measurement were prepared. *n* = 6 to 8 in each group. * *p* < 0.05, ** *p* < 0.01 as calculated by Kruskal-Wallis with Mann-Whitney post hoc analysis.

Additionally, we used Ellman’s reagent and kinetic assay to measure the glutathione content in the heart homogenates. As expected, GSH and GSSG levels were decreased by 2.2- and 3.8-fold in I/R group, compared to the control group (Figure 4). PAG + L-cysteine significantly increased GSH and GSSG levels by 3.5- and 2.1-fold respectively (*p* < 0.05 for both). In PAG + L-cysteine + I/R group, GSH and GSSG levels were decreased as well. However, they were significantly higher than in I/R group by 3.3- and 3.6-fold (*p* < 0.01 for both). BSO prevented PAG + L-cysteine mediated increase of GSH and GSSG levels in cardiac tissue by 71 (*p* < 0.01) and 30% respectively. As a result, the levels of GSH and GSSG in BSO + PAG + L-cysteine + I/R group were significantly lower than in PAG + L-cysteine + I/R hearts and reminded the values in I/R group. Thus, glutathione appears to be the main trigger in PAG + L-cysteine-mediated cardioprotection.

### 3.4. PAG + L-Cysteine Pretreatment Prevented I/R-Induced Oxidative and Nitrosative Stress

I/R caused the 2,4-fold increase in diene conjugates indicating intensified lipid peroxidation in I/R cardiac samples (Table 1). Notably, H_2_S level has significantly increased due to increased H_2_S synthase activity (CSE + CBS)in I/R group. The activity of cNOS and NO_2_^−^ level were 5.5- and 2.7-fold decreased that was in agreement with notable elevation of EDP during reperfusion and myocardial contraction in I/R group (Figure 2b). Simultaneous increase in iNOS activity and superoxide anion (˙O_2_^−^) generation rate resulted in double increase of nitrate anion (NO_3_^−^)—the product of peroxinitrite decomposition. Thus, I/R induced both oxidative and nitrosative stress.

PAG + L-cysteine + I/R hearts showed significantly lower generation rate of ˙O_2_^−^ and content of dien conjugates compared to I/R hearts. cNOS activity was increased under PAG + L-cysteine pretreatment (Table 1). Additionally, there was neither iNOS activity increase nor NO_3_^−^ levels elevation. Thus, one of the possible mechanisms of PAG + L-cysteine mediated cardioprotection may lie in effective prevention of ROS and reactive nitrogen species (RNS) production, preservation of NO synthesis and cNOS coupling.

### 3.5. PAG + L-Cysteine Pretreatment Inhibited Fatty Acids β-oxidation in Ischemized Heart

**Figure 5:**
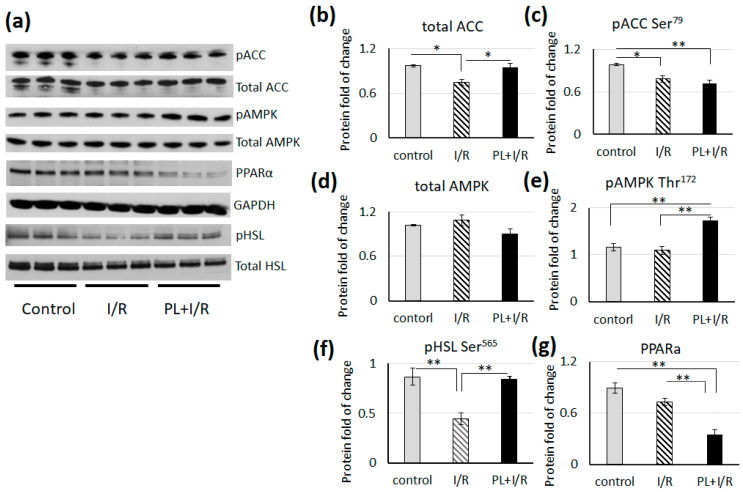
PAG+ L-cysteine pretreatment inhibitedfatty acids β-oxidation in I/R hearts. (**a**) Western blot of pACC at Ser79, ACC, pAMPK at Thr172, total AMPK, PPARa, pHSL at Ser565 andtotal HSL in heart lysates from control, I/R and PAG + L-cysteine + I/R (PL + I/R) groups. (Original western blot see supplementary Figure S2) (**b**)Densitometry of total ACC normalized to GAPDH. (**c**) Densitometry of pACC at Ser79 normalized to total ACC. (**d**)Densitometry of total AMPK normalized to GAPDH. (**e**) Densitometry of pAMPK at Thr172 normalized to total AMPK. (**f**)Densitometry of pHSL at Ser565normalized to total HSL. (**g**)Densitometry of total PPARa normalized to GAPDH. The data are expressed as the mean ± SEM of arbitrary fold of change relative to control levels. *n* = 6 to 8 per group. The control hearts were perfused by Langendorff preparation about 20 min, I/R heart underwent 20 min of global ischemia followed by 40 min of reperfusion. Then, lysates for WB were prepared. * *p* < 0.05, ** *p* < 0.01 (one-way ANOVA with post hoc Tukey HSD test).

Cardiac function under stress conditions is strongly dependent on metabolic plasticity for substrate utilization. Considering the improvement of the heart function after PAG + L-cysteine pretreatment, we focused on detailed analysis of the key proteins and signaling pathways that orchestrated cardiac metabolism. We performed WB analysis of lysates of hearts after I/R and PAG + L-cysteine + I/R treatment as well as the control hearts that were perfused for 20 min at Langendorff apparatus. The level of total ACC decreased in rat heart after I/R, at the same time pretreatment by PAG + L-cysteine preserved the level of total ACC upon reperfusion (Figure 5). However, the level of phosphorylated, thus inhibited, ACC was lower in the both groups of treated rats and this was more pronounced in heart lysates of PAG + L-cysteine + I/R group. The level of PPARa, one of the master regulators of lipid catabolism, was significantly lower in PAG + L-cysteine + I/R group compared to the control or to I/R group (Figure 5). These data indicate inhibition of fatty acids (FA) β-oxidation due to I/R but PAG + L-cysteine enhanced this effect.

Another important protein involved in regulation of energy metabolism - AMPK is known to be activated during hypoxia. However, its phosphorylation decreased to initial level after reoxygenation. We did not observe changes in total AMPK (Figure 5), however the level of phosphorylated AMPK was significantly increased in PAG + L-cysteine + I/R hearts (Figure 5). The level of pHSL at Ser 565, which is physiological target of AMPK, was significantly higher in heart lysates after PAG + L-cysteine treatment compared to I/R group (Figure 5), which additionally indicates the activation of AMPK. These data suggest that PAG + L-cysteine pretreatment has an inhibitory effect at FA β-oxidation in heart.

### 3.6. PAG + L-cysteinepretreatment Stimulated Glucose Consumption and Anaerobic Glycolysis in Ischemized Heart

**Figure 6:**
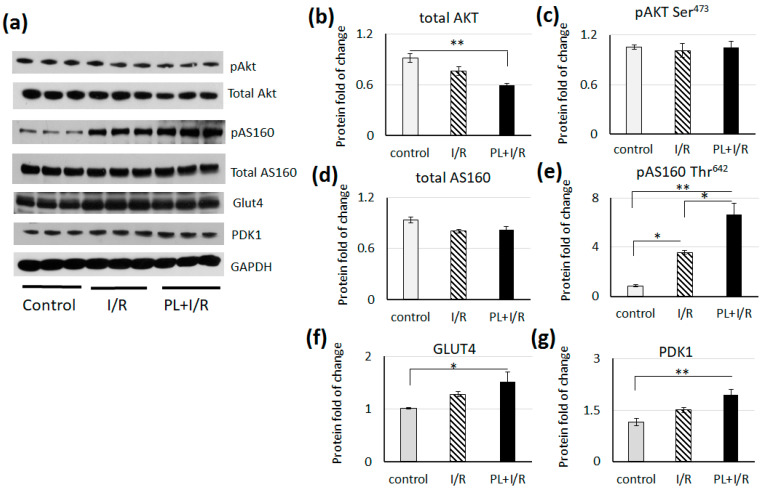
PAG+ L-cysteine pretreatment promoted glucose consumption and glycolysis in I/R hearts. (**a**) Western blot ofpAKT at Ser 473, total AKT, pAS160 atThr642, total AS160,GLUT4 and PDK1 in whole heart lysates from control, I/R and PAG + L-cysteine + I/R rats. (Original western blot see supplementary Figure S3) (**b**) Densitometry of total AKT normalized to GAPDH. (**c**) Densitometry of pAKT at Ser473 normalized to total AKT. (**d**) Densitometry of total AS160 normalized to GAPDH. (**e**) Densitometry of pAS160 at Thr642 normalized to total AS160. (**f**) Densitometry of GLUT4 normalized to GAPDH. (**g**) Densitometry of total PDK1 to GAPDH. The data are expressed as the mean ± SEM of arbitrary fold of change relative to control levels. *n* = 6 to 8 per group. Control hearts were perfused by Langendorff preparation about 20 min, I/R heart underwent 20 min of global ischemia followed by 40 min of reperfusion. Then, lysates for WB were prepared. * *p* < 0.05, ** *p* < 0.01 (one-way ANOVA with post hoc Tukey HSD test).

We examined the levels and activity of the main signaling pathways and enzymes involved in the glucose metabolism in heart. We did not observe the difference in pAKT at Ser 473 level in treated rat hearts, however the level of total AKT was significantly lower in hearts of PAG + L-cysteine + I/R group (Figure 6). The level of phosphorylated AS160 increased in both groups of treated rat hearts. But the level of pAS160 was significantly higher in heart lysates of PAG + L-cysteine + I/R group (Figure 6). Increase in pAS160 level is consistent with AMPK activity (Figure 5). As it was shown, AMPK is able to phosphorylate the AS160 in muscle cells and promotes glucose consumption [37]. This is in line with higher level of GLUT4, which was significantly higher in hearts of PAG + L-cysteine + I/R group (Figure 6). The level of PDK1, which phosphorylates and inactivates PDH, was significantly higher in PAG + L-cysteine + I/R group (Figure 6). This data indicates increase in glucose consumption, glycolysis and inhibition of oxidative glucose metabolism in PAG + L-cysteine pretreated hearts.

**Figure 7:**
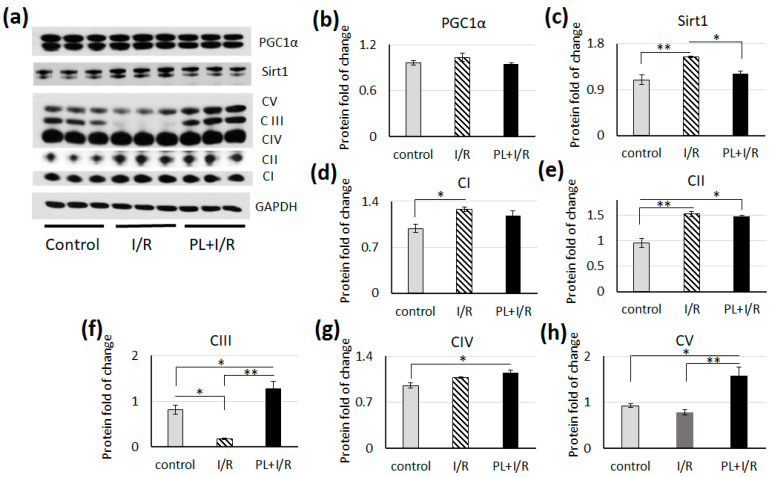
PAG + L-cysteine pretreatment increased key subunits of OXPHOS complex in I/R hearts. (**a**) Western blot of total PGC-1α, total Sirt1, complex I (C I), complex II (C II), complex III (C III), complex IV (C IV) and complex V (C V) whole heart lysates from the control, I/R and PAG + L-cysteine + I/R rats. (Original western blot see supplementary Figure S4) (**b**) Densitometry of total PGC-1α normalized to GAPDH. (**c**) Densitometry of total Sirt1 normalized to GAPDH. (**d**) Densitometry of Complex I normalized to GAPDH. (**e**) Densitometry of Complex II normalized to GAPDH. (**f**) Densitometry of Complex III normalized to GAPDH. (**g**) Densitometry of Complex IV normalized to GAPDH. (**h**) Densitometry of Complex V normalized to GAPDH. The data are expressed as the mean ± SEM of arbitrary fold of change, relative to control levels. *n* = 6 to 8 per group. The control hearts were perfused by Langendorff preparation about 20 min, I/R heart underwent 20 min of global ischemia followed by 40 min of reperfusion. Then, lysates for WB were prepared. * *p* < 0.05, ** *p* < 0.01 (one-way ANOVA with post hoc Tukey HSD test).

We did not observe changes in PGC-1α level, the main transcription factor governing mitochondrial biogenesis, in I/R and PAG + L-cysteine + I/R hearts (Figure 7). The level of SIRT1, which deacetylates and activates PGC-1α, was increased only in hears after I/R, while pretreatment with PAG + L-cysteine prevented this up-regulation (Figure 7).

Analysis of OXPHOS proteins revealed significantly increased levels of complexes I and II in I/R hearts, while the level of complexes III was significantly lower compared to the control (Figure 7). Pretreatment with PAG + L-cysteine stimulated protein expression of complexes II, III, IV and V compared to the control group. It is worth to note that PAG + L-cysteine pretreatment was accompanied by significant increase in the levels of complexes III and V compared to I/R group.

### 3.7. mTOR, MAPK/Erk1/2 and Canonical Wnt Signaling

Heart remodeling during reperfusion injury is governed by many signaling pathways including AMPK, canonical Wnt, cAMP/PKA, Pi3K–Akt and MAPK/Erk1/2 signaling pathways. The above-mentioned signaling pathways involved in cardiac performance as well as in cardiac metabolism regulation. Hence, we have examined their activity in the hearts after I/R and in hearts with PAG + L-cysteine pretreatment.

**Figure 8:**
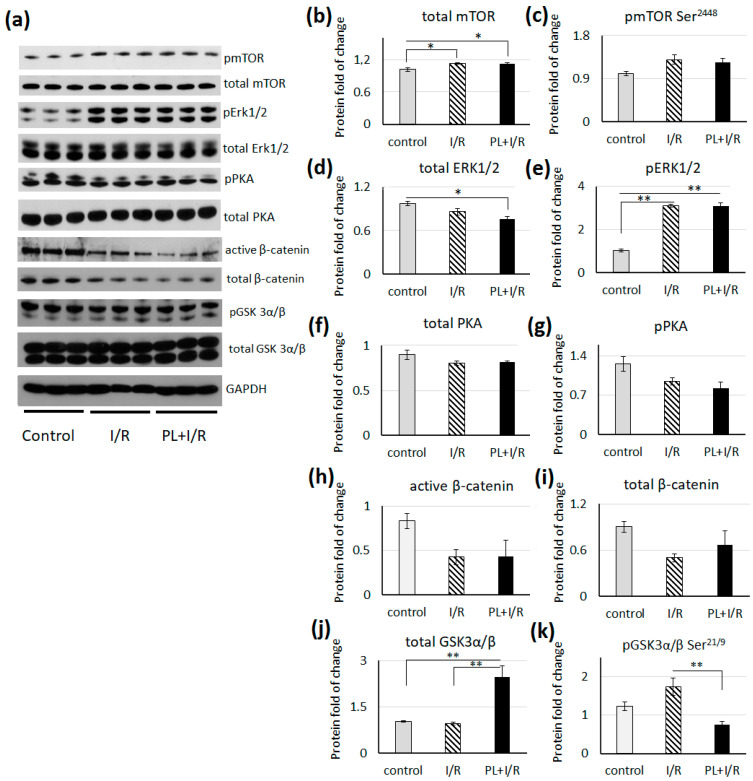
PAG + L-cysteine pretreatment does not affect mTOR, MAPK/Erk1/2 and canonical Wnt signaling activity in the I/R hearts. (**a**) Western blot of pmTOR at Ser 2448, total mTOR, pErk1/2, total Erk1/2, pPKA, total PKA, active β-catenin, total β-catenin, pGSK3β at Ser21/9 and total GSK3β in whole heart lysates from the control, I/R and PAG + L-cysteine + I/R rats. (Original western blot see supplementary Figure S5) (**b**) Densitometry of total mTOR normalized to GAPDH. (**c**) Densitometry of pmTOR at Ser2448 normalized to mTOR. (**d**) Densitometry of total Erk1/2 normalized to GAPDH. (**e**) Densitometry of pErk1/2 normalized to total Erk1/2. (**f**) Densitometry of total PKA normalized to GAPDH. (**g**) Densitometry of pPKA normalized to total PKA. (**h**) Densitometry of activeβ-catenin normalized to total β-catenin. (**i**) Densitometry of total β-catenin normalized to GAPDH. (**j**) Densitometry of total GSK3α/β normalized to GAPDH. (**k**) Densitometry of pGSK3α/βatSer21/9 normalized to total GSK3α/β. The data are expressed as the mean ± SEM of arbitrary fold of change relative to control levels. *n* = 6 to 8 per group. Control hearts were perfused by Langendorff preparation about 20 min, I/R heart underwent 20 min of global ischemia followed by 40 min of reperfusion. Then, lysates for WB were prepared. * *p* < 0.05, ** *p* < 0.01 (one-way ANOVA with post hoc Tukey HSD test).

The level of total mTOR was significantly higher in I/R and PAG + L-cysteine hearts compared to the control, while level of phosphorylated mTOR was unchanged in both groups (Figure 8). We should note that this phosphorylation at Ser2448 is facilitated by AKT, however, in our experiment we did not register the pAKT level increase in both groups of treated hearts (Figure 6). The level of total ERK1/2 was significantly lower in PAG + L-cysteine + I/R group compared to the control (Figure 8). The levels of phosphorylated ERK1/2 protein has significantly increased in both groups of treated rats, however there have been registered no difference between I/R hearts and hearts of PAG + L-cysteine + I/R group. We did not observe changes in the level of total and phosphorylated PKA as well as total and activated b-catenin (Figure 8). However, the level of total GSK3b was significantly higher and the level of phosphorylated GSK3b was significantly lower in hearts of PAG + L-cysteine + I/R group (Figure 8).

## 4. Discussion

I/R is associated with numerous biochemical and molecular changes including increase in ROS production, glutathione depletion and NOS uncoupling which negatively affects cells survival and heart function [38]. In this study, stimulation of endogenous glutathione synthesis provided cardioprotection against I/R in rats. We used glutathione precursor, L-cysteine, and an inhibitor of its conversion to H_2_S, PAG, thus, directing exogenous and/or endogenous L-cysteine for glutathione synthesis. Our data showed that simultaneous treatment with PAG and L-cysteine significantly increased GSH and GSSG levels in cardiac tissue in normal condition and upon I/R. The use of glutathione inhibitor, BSO, has completely abolished the cardioprotective effect of PAG + L-cysteine, preventing increase of glutathione levels. The main features of PAG + L-cysteine cardioprotection were manifested as unchanged myocardial relaxation and the oxygen cost of myocardial work during reperfusion compared to pre-ischemic state. Biochemical data supported the physiological ones and showed antioxidative effect of PAG + L-cysteine combination as decrease in ROS production and preservation of cNOS activity in the heart upon I/R. We find it interesting that both H_2_S synthesis inhibitor and mitochondrial targeted H_2_S donor exert protective effect in a mouse model of burn injury reducing changes in inflammatory cytokines and organ injury markers [39]. Thus, our data is in a line with observations of others about complex role of H_2_S in pathologic conditions.

Additional evidence of cardioprotection due of PAG + L-cysteine pretreatment came from Western blot analysis of proteins involved in cardiac energetic metabolism and mitochondria function. PAG + L-cysteine pretreatment enhanced glucose consumption and glycolysis in I/R heart as evidenced by increase in the level of GLUT4, PDK1 and pAS160. Crosstalk between metabolic shift and mitochondrial ROS production was shown recently. Similarly to our work, Beltran et al. demonstrated that activation of glycolysis during I/R injury reduced ROS production [20]. The FA β-oxidation also is associated with ROS production. Thus, reduced lipids catabolism rate is accompanied by lower level of ROS. We found that PAG + L-cysteine inhibited FA β-oxidation in the heart, since we observed the lower levels of pACC and PPARa in PAG + L-cysteine + I/R group. Moreover, metabolic shift from FA β-oxidation to glucose utilization reduced the oxygen cost of myocardial work by 11–13% and was cardioprotective [40,41]. We should note that we observed significant decrease in the oxygen cost of myocardial work in the injured heart after PAG + L-cysteine pretreatment. This observation is in a line with published data [40,41] and suggests that PAG + L-cysteine promote metabolic shift to anaerobic metabolism which resulted in the decrease of ROS as well as optimization of oxygen use in the injured heart.

As it was mentioned, reduced glutathione is crucial for proper mitochondria functioning, thus stimulation of glutathione synthesis by PAG + L-cysteine might positively affected mitochondria function. PAG + L-cysteine induced significant elevation in the levels of OXPHOS complexes II, III, IV and V which indicate enhanced rate of electron transport, mitochondrial Ca^2+^ influxand, probably, ATP production in the injured heart. Increased OXPHOS proteins together with increased SERCA2 level evidence for sufficient ATP supply and effortless Ca^2+^ re-entry into sarcoplasmic reticulum in the injured heart after PAG + L-cysteine pretreatment. This supports the improved diastolic function of the isolated rat heart in PAG + L-cysteine + I/R group. The level of PGC-1α, the key regulator of mitochondrial biogenesis was unchanged in I/R and PAG + L-cysteine + I/R hearts. It is quite interesting that the level of Sirt1, which promote mitochondria biogenesis via PGC-1α pathway [42] has elevated in I/R hearts and normalized to the control level in the case of PAG + L-cysteine pretreatment. Although our data indicate changes in mitochondrial electron transport proteins under PAG + L-cysteine pretreatment, further studies of mitochondrial respiration and membrane potential are needed to understand the function changes in energy production.

Our data clearly demonstrated that PAG + L-cysteine pretreatment modulates a few signaling pathways: we observed activation of AMPK and inhibition of PPARa signaling pathways. Additionally, we have found lowering in pGSK3b which has dozens of functions in the cell. All these signaling involved in heart remodeling and cardiac metabolism regulation. The activation of AMPK might occur via in vitro glutathionylation by two mammalian glutathione-S-transferase isoforms [43]. During stress conditions activation of AMPK, by ROS or RNS for example, force energy production in injured cardiomyocytes by modulation of substrate metabolism (i.e., glucose uptake increase) [44] via AS160 phosphorylation [37] what allow GLUT4 translocation into the cells membrane [45]. We supposed that PAG + L-cysteine similarly to aspirin [46] and carvedilol [44] protects against reperfusion injury through AMPK signaling. However, our data also showed decrease in the level of master regulator of lipids metabolism—PPARa in PAG + L-cysteine + I/R hearts which is consistent with inhibited FA β-oxidation and up-regulated glucose utilization in I/R hearts, pretreated with PAG + L-cysteine.

## 5. Conclusions

In this work we have shown that simultaneous application of H_2_S precursor (L-cysteine) and inhibitor of CSE (D,L-propargylglycine) has a strong cardioprotective effect against reperfusion injury. This combination attenuated I/R-induced ROS and RNS production and prevented cNOS uncoupling. We demonstrated that this effect was mediated by L-cysteine derivate, glutathione. An inhibitor of glutathione synthesis, DL-buthionine-(S,R)-sulfoximine, abolished cardioprotective effect caused by PAG + L-cysteine pretreatment. We have found that PAG + L-cysteine + I/R hearts displayed decrease in FA β-oxidation; increase in glucose consumption; glycolisys; OXPHOS proteins; and preserved SERCA2 levels. Possible protective action of PAG + L-cysteine treatment in I/R heart is realized via AMPK signaling pathway activation as well as PPARa inhibition, which govern cardiomyocytes metabolism and metabolic plasticity under the I/R conditions. Our results do not reduce the functional value of H_2_S in the heart but introduce the novel aspect of crosstalk between metabolic pathways of L-cysteine. Thus, PAG + L-cysteine is a promising combination for endogenous glutathione stimulation in case of oxidative stress.

## Figures and Tables

**Table 1:** Biochemical indexes of rat cardiac tissue.

Index	Control (*n* = 8)	I/R (*n* = 10)	PAG + L-cysteine (*n* = 5)	PAG + L-cysteine + I/R (*n* = 7)
O_2_^−^, nmol mg^−1^ min^−1^	2.63 ± 0.08	8.72 ± 0.57 ***	1.61 ± 0.05 ***	2.37 ± 0.11 ^###^
H_2_O_2_, pmol mg^−1^	0.79 ± 0.04	2.27 ± 0.26 ***	0.69 ± 0.02	1.15 ± 0.11 ^##^
Diene conjugates, ng mg^−1^	3.59 ± 0.25	8.84 ± 0.48 ***	0.92 ± 0.12 ***	5.24 ± 0.63 ^###^
cNOS activity, pmol mg^−1^ min^−1^	7.51 ± 0.19	1.36 ± 0.09 ***	8.57 ± 0.30 *	2.38 ± 0.34 ^##^
iNOS activity, pmol mg^−1^ min^−1^	2.64 ± 0.15	7.07 ± 0.20 ***	1.68 ± 0.04 ***	3.86 ± 0.26 ^###^
NO_2_^−^, pmol mg^−1^	361.8 ± 17.9	131.5 ± 12.2 ***	440.1 ± 5.2 **	343.4 ± 5.17 ^###^
NO_3_^−^, nmol mg^−1^	10.92 ± 0.21	22.27 ± 0.88 ***	5.24 ± 0.13 ***	9.95 ± 0.40 ^###^
H_2_S, pmol mg^−1^	17.56 ± 1.26	96.80 ± 7.90 ***	14.56 ± 0.77	29.23 ± 0.86 ^###^
CSE + CBS activity, pmol of H_2_S mg^−1^ min^−1^	8.80 ± 0.22	30.15 ± 2.95 ***	7.71 ± 0.11 **	16.48 ± 0.81 ^##^

Control hearts were perfused by Langendorff preparation about 20 min. * *p* < 0.05, ** *p* < 0.01, *** *p* < 0.001 versus control, ^##^ *p* < 0.01, ^###^ *p* < 0.001 versus I/R as calculated by one-way ANOVA with post hoc Tukey HSD test.

## Data Availability

Data are contained within the article.

## Supplementary Materials

The following are available online at https://www.mdpi.com/article/10.3390/life11070631/s1, Figure S1: Raw data of Western blots used on Figure 3a. Figure S2: Raw data of Western blots used on Figure 5a. Figure S3: Raw data of Western blots used on Figure 6a. Figure S4: Raw data of Western blots used on Figure 7a. Figure S5: Raw data of Western blots used on Figure 8a.
