# Supplementary material for: Induction of Glutathione Synthesis Provides Cardioprotection Regulating NO, AMPK and PPARa Signaling in Ischemic Rat Hearts
Source: Life (Basel). 2021 Jun 29;11(7):631. doi: 10.3390/life11070631 (PMC8308105; doi:10.3390/life11070631)
Supplement: Supplementary file 1 [file life-11-00631-s001.zip › life-1244691-supplementary.pdf]

life

MDPI

Article

Induction of Glutathione Synthesis Provides Cardioprotection Regulating NO, AMPK and PPARa Signaling in Ischemic Rat Hearts

Yulia V. Goshovska [1,*], Raisa A. Fedichkina [1], Volodymyr V. Balatskyi [2], Oksana O. Piven [3], Pawel Dobrzyn [2] and Vadym F. Sagach [1]

[1] Department of Blood Circulation, Bogomoletz Institute of Physiology, National Academy of Sciences of Ukraine, 4 Bogomolets Str., Kyiv 01024, Ukraine; goshovska@biph.kiev.ua (Y.V.G.); fedichkina@biph.kiev.ua (R.A.F.); vsagach@biph.kiev.ua (V.F.S.)

[2] Laboratory of Molecular Medical Biochemistry, Nencki Institute of Experimental Biology, Polish Academy of Sciences, 3 Pasteur Str., 02-093 Warsaw, Poland; v.balatskyi@nencki.edu.pl (V.V.B.); p.dobrzyn@nencki.edu.pl (P.D.)

[3] Department of Human Genetics, Institute of Molecular Biology and Genetics, National Academy of Sciences of Ukraine, 150 Akad. Zabolotnogo Str., Kyiv 03680, Ukraine; o.o.piven@imbg.org.ua

* Correspondence: goshovska@biph.kiev.ua; Tel.: +380-442562485; Fax: +380-442562000

Abstract: Glutathione (GSH) is essential for antioxidant defence, and its depletion is associated with tissue damage during cardiac ischemia-reperfusion (I/R). GSH is synthesized by the glutamate-cysteine ligase enzyme (GCL) from L-cysteine, which alternatively might be used for hydrogen sulfide production by cystathionine-gamma-lyase (CSE). Here, we have investigated whether in vivo treatment with L-cysteine and an inhibitor of CSE, D,L-propargylglycine (PAG), can modulate cardiac glutathione and whether this treatment can influence heart resistance to I/R in a Langendorff isolated rat hearts model. Pretreatment with PAG+L-cysteine manifested in pronounced cardioprotection, as there was complete recovery of contractile function; preserved constitutive NOS activity; and limited the production of reactive oxygen and nitrogen species in the ischemized myocardium. Cardiac GSH and GSSG levels were increased by 3.5- and 2.1-fold in PAG+L-cysteine hearts and were 3.3- and 3.6-fold higher in PAG+L-cysteine+I/R compared to I/R heart. The cardioprotective effect of PAG+L-cysteine was completely abolished by an inhibitor of GCL, DL-buthionine-(S,R)-sulfoximine. Further analysis indicated diminished fatty acid β-oxidation, increased glucose consumption and anaerobic glycolysis,and promoted OXPHOS proteins and SERCA2 in PAG+L-cysteine+I/R compared to the I/R group. PAG+L-cysteine inhibited PPARα and up-regulated AMPK signalling in the heart. Thus, induction of glutathione synthesis provided cardioprotection regulating NO, AMPK and PPARa signaling in ischemic rat hearts.

Keywords:cardioprotection; glutathione; ischemia; heart; SERCA; AMPK; PPARα

Citation: Goshovska, Y.V.; Fedichkina, R.A.; Balatskyi, V.V.; Piven, O.O.; Dobrzyn, P.; Sagach, V.F. Induction of Glutathione Synthesis Provides Cardioprotection Regulating NO, AMPK and PPARa Signaling in Ischemic Rat Hearts. *Life* **2021**, *11*, 631. https://doi.org/10.3390/life11070631

Academic Editor: Friedrich Jung

Received: 17 May 2021
Accepted: 11 June 2021
Published: 29 June 2021

Publisher's Note: MDPI stays neutral with regard to jurisdictional claims in published maps and institutional affiliations.

Copyright: © 2021 by the authors. Submitted for possible open access publication under the terms and conditions of the Creative Commons Attribution (CC BY) license (http://creativecommons.org/licenses/by/4.0/).

Supplementary Materials:

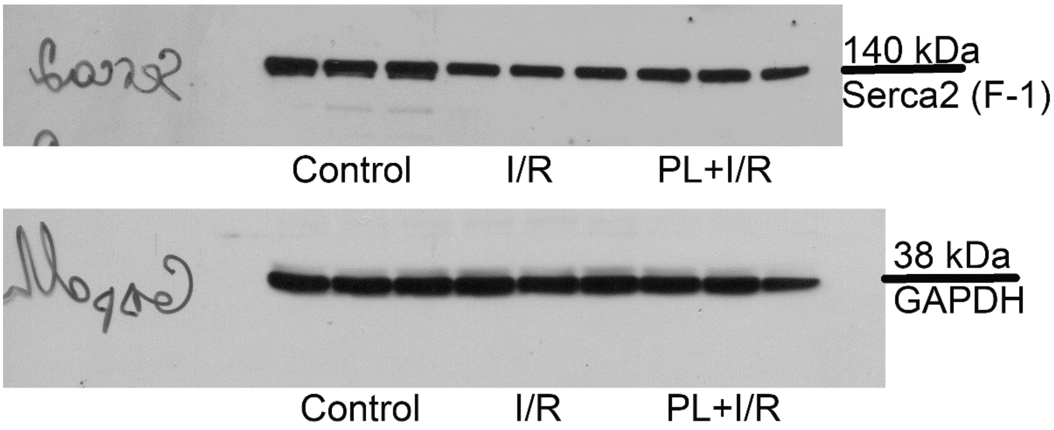

Figure S1. Raw data of Western blots used on Figure 3a.

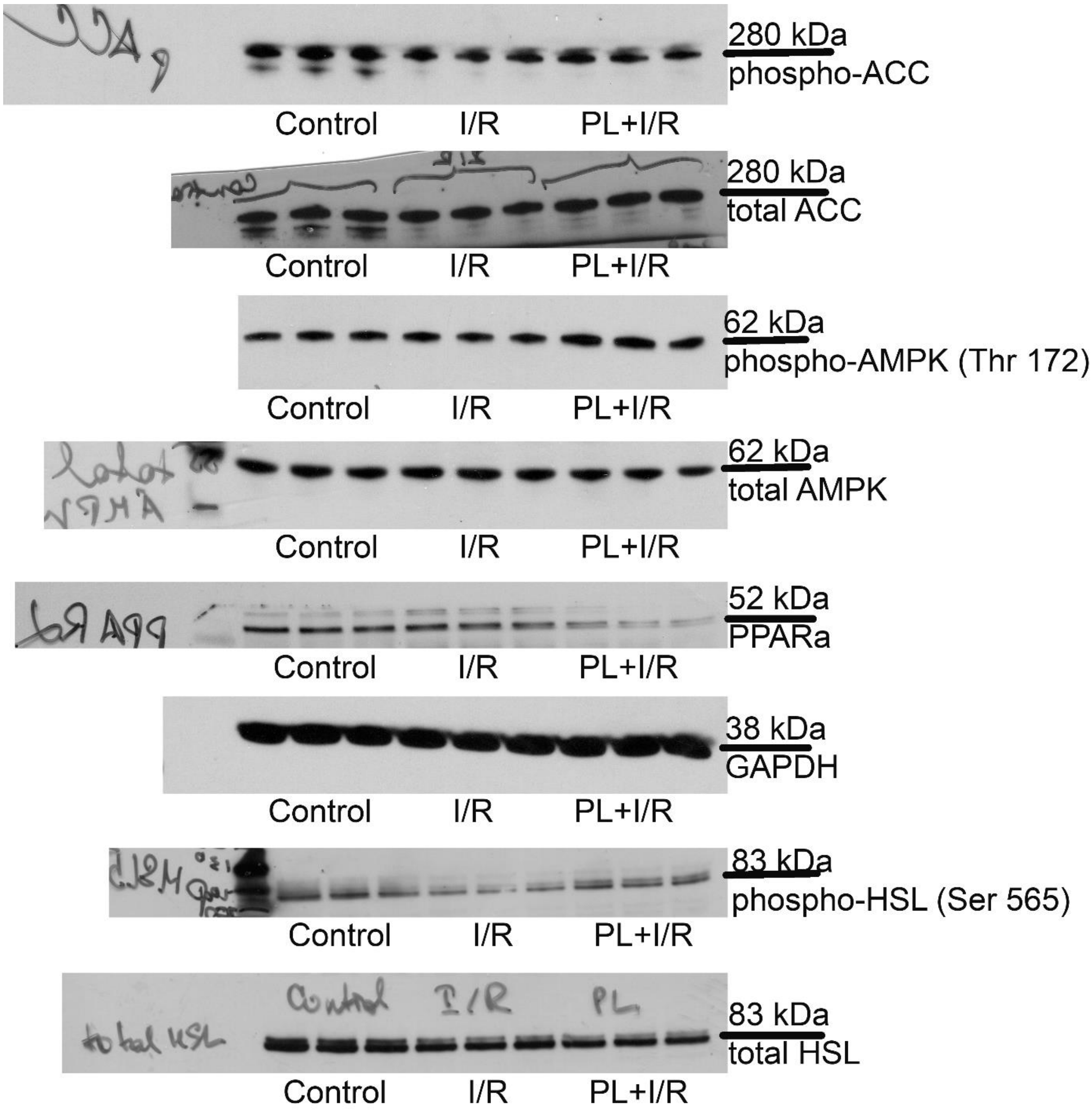

Figure S2. Raw data of Western blots used on Figure 5a.

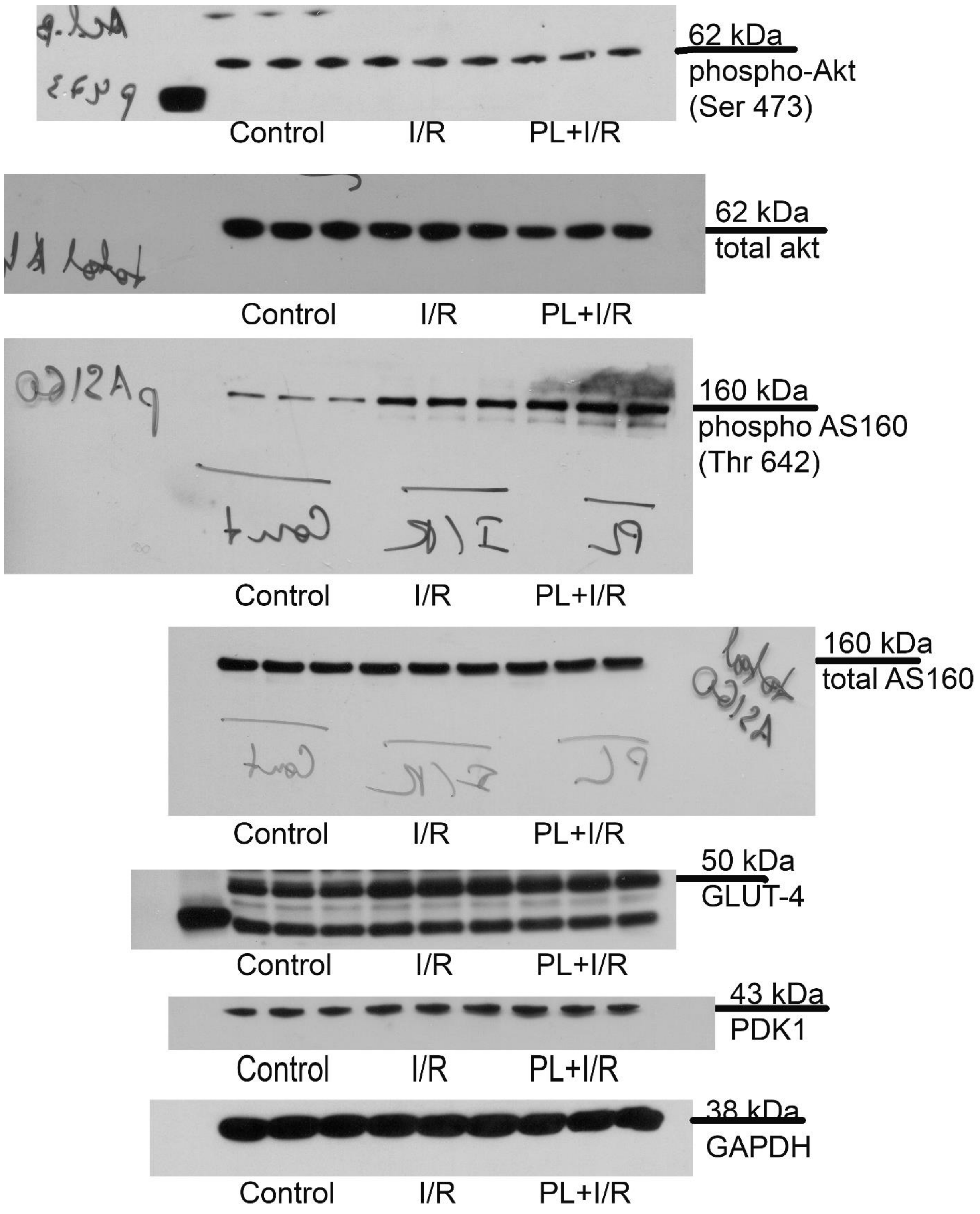

Figure S3. Raw data of Western blots used on Figure 6a.

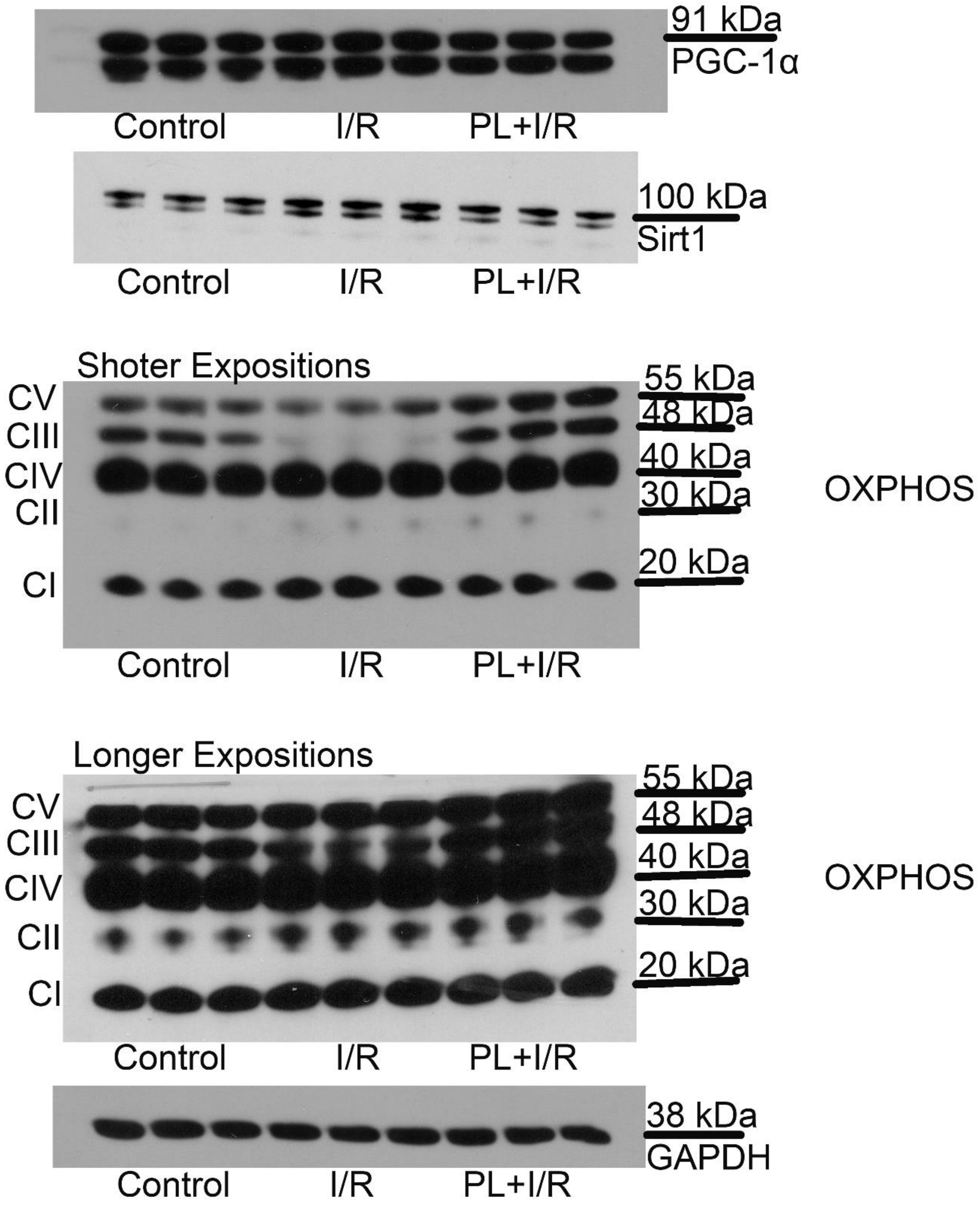

Figure S4. Raw data of Western blots used on Figure 7a.

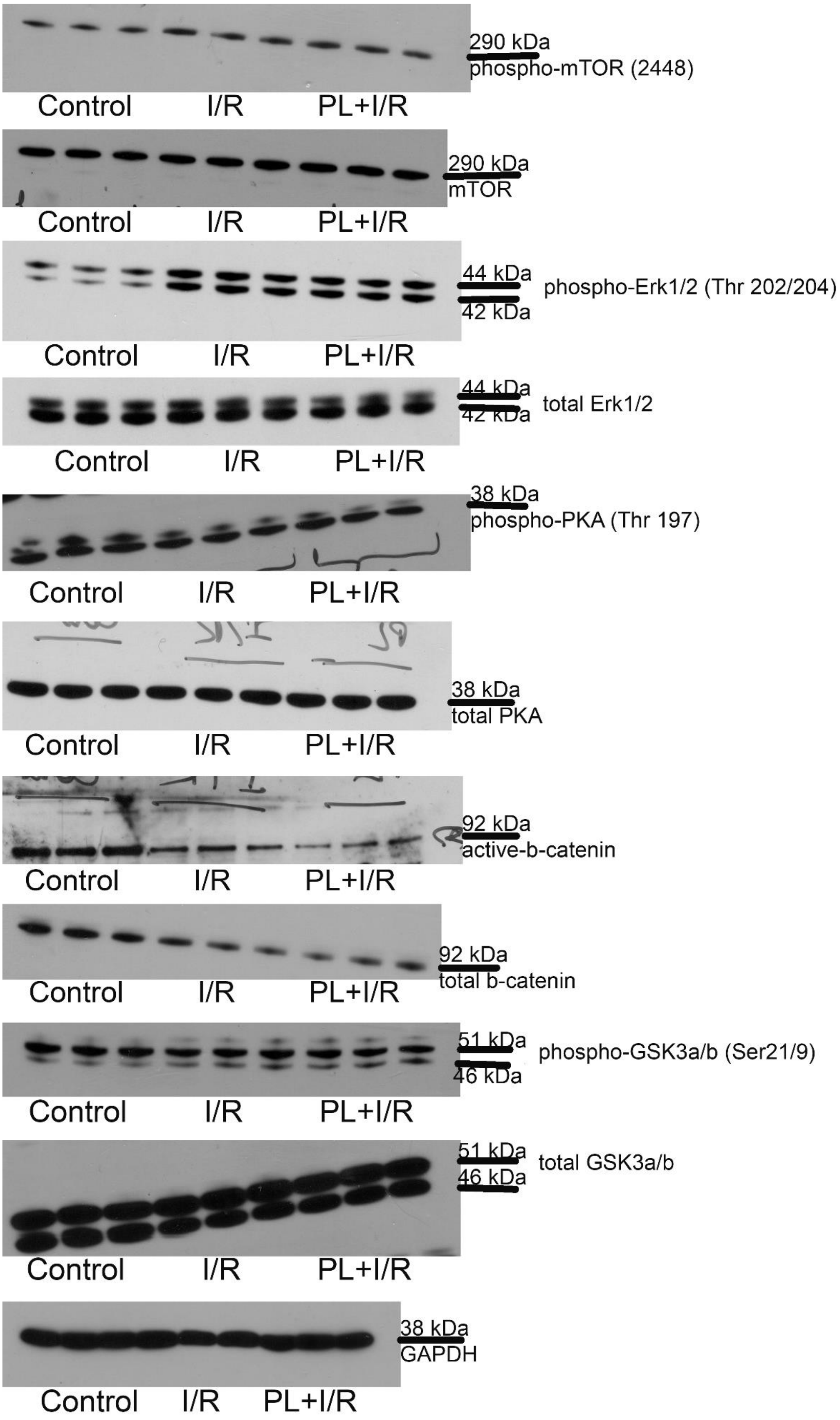

Figure S5. Raw data of Western blots used on Figure 8a.
